# Platelet-Membrane-Camouflaged Zirconia Nanoparticles Inhibit the Invasion and Metastasis of Hela Cells

**DOI:** 10.3389/fchem.2020.00377

**Published:** 2020-05-07

**Authors:** Yinghui Shang, Qinghai Wang, Jian Li, Qiangqiang Zhao, Xueyuan Huang, Hang Dong, Haiting Liu, Rong Gui, Xinmin Nie

**Affiliations:** ^1^Department of Blood Transfusion, the Third Xiangya Hospital, Central South University, Changsha, China; ^2^Department of Cardiology, the Second Hospital of Shandong University, Jinan, China; ^3^Clinical Laboratory of the Third Xiangya Hospital, Central South University, Changsha, China

**Keywords:** platelet membrane, zirconia, invasion, metastasis, anticancer

## Abstract

Zirconia nanoparticles (ZrO2 NPs) are widely applied in the field of biomedicine. In this study, we constructed a nanoplatform of ZrO2 NPs coated with a platelet membrane (PLTm), named PLT@ZrO2. PLTm nanovesicles camouflage ZrO2 NPs, prevent nanoparticles from being cleared by macrophage, and target tumor sites. Compared to ZrO2 alone, PLT@ZrO2 is better at inhibiting the invasion and metastasis of Hela cells *in vitro* and *in vivo. In vitro*, PLT@ZrO2 inhibited the growth and proliferation of Hela cells. Scratch-wound healing recovery assay demonstrated that PLT@ZrO2 inhibited Hela cells migration. Transwell migration and invasion assays showed that PLT@ZrO2 inhibited Hela cells migration and invasion. *In vivo*, PLT@ZrO2 inhibited the tumor growth of Xenograft mice and inhibited the lung and liver metastasis of Hela cells. Immunofluorescence and Western blotting results showed that anti-metastasis protein (E-cadherin) was upregulated and pro-metastasis proteins (N-cadherin, Smad4, Vimentin, E-cadherin,β-catenin, Fibronectin, Snail, Slug, MMP2, Smad2) were down-regulated. Our study indicated that PLT@ZrO2 significantly inhibits tumor growth, invasion, and metastasis.

## Introduction

Zirconium oxide nanoparticles (ZrO2 NPs) possess many good electrochemical properties, such as non-toxicity, thermal stability, wide band gap, and excellent electrical and surface performances (Yang et al., 2007). ZrO2-based biosensors for oral cancer drug detection (Kumar et al., 2016), and electrochemical sensors for anticancer drugs by ZrO2 NPs-decorated nanocomposite (Venu et al., 2018), have also been implemented. Sulphated zirconia nanoparticles have been studied in anticancer applications (Mftah et al., 2015) and sulfated zirconia nanoparticles doped by iron-manganese are cytotoxic to cancer cells (Al-Fahdawi et al., 2015). Moreover, ZrO2 NPs have exhibited cytotoxicity against human carcinoma cell lines (Balaji et al., 2017), but their antitumor activity and mechanism has not been explored thoroughly.

Current studies have indicated that tumor metastasis is facilitated by blood platelets (PLTs) (Karpatkin and Pearlstein, 1981; Gasic, 1984; Tanaka et al., 1986; Chen et al., 1992; Honn and Tang, 1992a,b; Nieswandt et al., 1999), and platelets and tumor cells are bound by P-selectin and the CD44 receptor (Borsig et al., 2001; Hu et al., 2015a) with capture based on structure (Sabrkhany et al., 2018). Previous studies have indicated that platelet-membrane-camouflaged black phosphorus quantum dots could target tumor sites (Shang et al., 2019), but it has not been studied whether platelet-membrane-camouflaged nanoparticles can target tumor metastasis.

Although surgical techniques and chemotherapy regimens have improved, metastasis remains a serious barrier to the effective treatment for patients with cervical cancer (Li et al., 2016), and the prognosis of patients suffered from metastatic cervical cancer is poor (Peng et al., 2016). To address these challenges, we constructed platelet-membrane-camouflaged ZrO2 NPs (PLT@ZrO2) to investigate targeting of metastatic tumors and anti-metastasis activity.

## Materials and Methods

### Materials

Zirconia nanoparticles (XF101) were prepared by the XFNANO Materials Tech Co., Ltd. (China). Yeasen Biotechnology (China) provided Cy5, Rhodamine B (RhB), Hoechst 33342, and distearyl phosphatidyl ethanolamine-fluorescein isothiocyanate (DSPE-FITC). Solarbio (China) provided dialysis membranes (2 kD). Whatman (USA) provided polycarbonate porous membrane syringe filters (200 nm). Life Technologies (USA) provided RPMI-1640, Dulbecco's modified Eagle medium (DMEM) (high glucose), fetal bovine serum (FBS), and trypsin EDTA. Servicebio Technology Co., Ltd. (China) supplied One Step TUNEL Apoptosis Assay Kit, DAPI, Calcein-AM, crystal violet, hematoxylin and eosin (HE), anti-N-cadherin, anti-Vimentin, anti-E-cadherin, anti-β-catenin, anti-Fibronectin, anti-Snail, anti-Smad4, anti-Slug, anti-MMP2, anti-Smad2, anti-β-actin, anti-GAPDH antibodies, horseradish peroxidase (HRP) goat anti-rabbit IgG secondary antibodies, cy3 goat anti-IgG and FITC goat anti-IgG secondary antibodies, and a prestained protein ladder.

### Cells and Animals

The Cancer Research Institute of Central South University gifted the Hela human cervical cancer cell line. The Hela cells were cultured in DMEM (high glucose) containing 10% FBS. RAW264.7 macrophages were cultured in RPMI-1640 medium containing 10% FBS. All cells were incubated at 37°C in a 5% CO_2_ incubator. Hunan SJA Laboratory Animal Co., Ltd provided 6 week old female Balb/c nude mice.

### PLTm Vesicles Preparation

PLTs were obtained by centrifugation (1,500 rpm, 5 min) and double washing with PBS of whole blood from female Balb/c nude mice. PLTm vesicles of ~150 nm were prepared by repeated freezing (at −80°C, 2 h) and thawing (at 37°C, 10 min) PLTs, and ultrasonic treatment (2 min, 42 kHZ, 100 W) of PLTs (Hu et al., 2015b).

### Construction of PLT@ZrO2

Ultrasonic treatment (5 min, 42 k Hz, 100 W) of ZrO2 NPs with an equal volume of PLTm vesicles promoted camouflage of the nanoparticles. After filtration with porous syringe filters (200 nm) and centrifugation (2,500 rpm, 10 min), PLTm vesicles which did not camouflage ZrO2 NPs were settled out and the PLT@ ZrO2 were obtained. Generally, 300 μL ZrO2 NPs (50 μg/mL) were mixed with 300 μL PLTm vesicles to pr2Eepare 300 μL PLT@ZrO2.

### Characterization of PLT@ZrO2

A transmission electron microscope (TEM) (Tecnai G2 Spirit, FEI, USA) was used to check the morphology and size of PLT@ZrO2. We could observe the size of the nanoparticles as well as identify whether the nanoparticles were encapsulated into PLTm vesicles. A silicon chip dipped in anhydrous ethanol containing PLT@ZrO2 was examined with atomic force microscopy (AFM) (MFP-3D-S, Asylum Research, USA) to detect the heights of nanoparticles. Zetasizer Nano ZS (Malvern Nano series, Malvern, U.K.) was used to measure the particle sizes and surface charges. UV/vis spectroscopy (ScanDrop, Analytik Jena, Germany) was applied to detect the absorbance of PLT@ZrO2.

### Cell Viability of Hela Cells Assessed Through Crystal Violet Staining

Hela cells were inoculated into 35 mm dishes (2 × 10^5^/dish). After 24 h, the cells were treated with new medium, ZrO2, PLTm vesicles or PLT@ZrO2. The concentration of ZrO2 NPs used in this study was 50 μg/mL. After 24 h, crystal violet 300 μl was added into each well and then washed with PBS to assess the viable cells.

### Scratch-Wound Healing Recovery Assays

Hela cells were seeded in a 6-well plate (1 × 10^5^/well). After 24 h, the culture medium was removed, straight incisions were made by a 10 μl pipette tip, and then cells were washed with PBS to remove detached and suspended cells. The remaining cells were given new medium, ZrO2, PLTm vesicles, or PLT@ZrO2, respectively. The concentration of ZrO2 NPs used in this study was 50 μg/mL. After 24 h, pictures of the scratches were taken under an inverted phase contrast microscope (Axio Observer, ZEISS, Germany), and the extent of wound healing recovery was assessed by the wound area in each group.

### Migration and Invasion Assays

Transwell Permeable Supports (Corning Inc., USA) were used to assay cell migration and invasion. Hela cells (about 1 × 10^4^/well) suspended in 200 μl serum-free medium with ZrO2, PLTm vesicles, or PLT@ZrO2, were plated onto Transwell filter inserts in 24-well plates for migration assays. The concentration of ZrO2 NPs used in this study was 50 μg/mL. The corresponding cell suspensions plated onto Transwell filter inserts coated with Matrigel were used for invasion assays. We used 500 μl DMEM containing 10% FBS plated in the lower chambers as a chemoattractant. After incubation for 24 h, a cotton swab was used to remove the cells in the upper chamber. Cells on the bottom side were stained with Calcein-AM (Jang et al., 2012) and photographed under an inverted fluorescence microscope at excitation wavelength of 530 nm and emission wavelength of 590 nm. Pictures were quantified and analyzed using MetaXpress software (Molecular Devices).

### Biocompatibility of PLT@ZrO2

Hemolytic rates and RAW 264.7 macrophages phagocytosis were conducted to estimate the biocompatibility of PLT@ZrO2. After incubating ZrO2 NPs or PLT@ZrO2 (from 0.1 to 0.8 mg/mL) with 5% red blood cell suspension at 37°C for 2 h, the mixture was centrifuged (3,500 rpm, 5 min), and the absorbance at 545 nm of the supernatant was measured by a microplate reader. Ultra-pure water and PBS incubated with 5% red blood cell suspension was used as a positive and negative control, respectively. We calculated the hemolytic ratio as follows: hemolytic rate% = (experimental sample absorbance—negative control absorbance)/(positive control absorbance—negative control absorbance) × 100%. RAW 264.7 macrophages (1 × 10^5^/well) were seeded into a 6-well plate and treated with 2 mL PLT@ZrO2-RhB or ZrO2-RhB for 24 h to assess the ability of PLT@ZrO2 to evade the immune response. In the nanocomposite, the concentration of ZrO2 NPs was 50 μg/mL. After staining the cell nuclei with Hoechst 33342 (Crowley et al., 2016), pictures taken from a laser confocal fluorescence microscope (LCFM) (TCS SP8 CARS, Leica, Germany) were used to assess the phagocytosis of PLT@ZrO2 by macrophages and the fluorescence intensity.

### PLT@ZrO2 Distribution Assay *In vivo*

Hela tumor-bearing mice were injected through the tail vein with the Cy5-labeled PLT@ZrO2 or Cy5-labeled ZrO2 to evaluate the targeting capability of PLT@ZrO2 *in vivo*. The dosage of Cy5 in the nanocomposite was 3 μg/kg. After administration, a Xenogen IVIS Lumina XR imaging system (Caliper Life Sciences, USA) was applied to detect the fluorescence intensity of the mice at 6, 24, and 48 h, respectively. The mice were then euthanized, and tumor tissues and visceral tissues were harvested for further imaging. Then the tumor tissues were made into frozen sections and observed under an inverted fluorescence microscope.

### PLT@ZrO2 Treatment in Cervical Cancer-Bearing Mice

To explore *in vivo* anticancer effect, we injected Hela cells (1 × 10^6^) in 100 μL PBS through the hypodermic and tail vein, to establish subcutaneous tumors and metastasis tumors, respectively. Tumor volume was calculated as follow: V = 1/2 × D × L^2^, where V refers to volume, D refers to the longitudinal diameter and L refers to the latitudinal diameter. Day 0 (D0) represents the first day when subcutaneous tumor volumes exceed 100 mm^3^. Fifteen mice were randomly assigned to 3 groups (*n* = 5) and injected with 100 μL of PBS, PLTm, or PLT@ZrO2 through tail vein once a day for 3 consecutive days. The dosage of ZrO2 was 50 mg/kg/d. The tumor sizes and body weights of the mice were measured, and tumor volumes were calculated once every 4 days. All mice were euthanized on day 14 (D14). Whole blood, tumors, and internal organs (hearts, livers, spleens, lung, and kidney) were collected. Whole blood was collected and measured by a five-part differential hematology analyzer (BC-5390, Mindray, China). After centrifugation (3,000 rpm, 10 min), an automatic biochemical analyzer (7100, HITACHI, Japan) and an immunology analyzer (Cobas 6000 e601, ROCHE, USA) were applied to detect the serum enzyme levels. All collected organs and tumors were fixed in 4% paraformaldehyde and frozen at −80°C. The frozen tumor tissues were used for Western blotting analysis. The fixed tissues were embedded in paraffin, sliced into sections, and then stained for HE and immunofluorescence.

### Immunofluorescence Analysis

Immunofluorescence analysis was performed by immunofluorescence staining of TUNEL, N-cadherin, Vimentin, E-cadherin, β-catenin, and Fibronectin according to standard protocols (Hseu et al., 2019). The sections were then counterstained with DAPI (Chazotte, 2011), observed under an inverted fluorescence microscope, and photographed.

### Western Blotting Analysis

Proteins were extracted from tumor tissue lysates using RIPA buffer. The concentrations of total proteins were quantified with a BCA protein assay kit. Protein expressions were assessed by immunoblot analysis of tumor tissue lysates (40 μg) in the presence of rabbit antibodies against Snail, Smad4, Vimentin, Slug, N-cadherin, MMP2, Smad2, GAPDH (1:1,000, Servicebio Technology, China), and mouse antibodies against E-cadherin and β-actin (1:1,000, Servicebio Technology, China), according to standard protocols (Hseu et al., 2019).

### Statistical Analysis

SPSS Software 20.0 was used for statistical analysis. Data are expressed as the mean ± SD. One-way ANOVA was used to assess the differences between groups, and Tukey's posttest was performed (^*^ indicates *p* < 0.05, ^**^ indicates *p* < 0.01, ^***^ indicates *p* < 0.001, and ^****^ indicates *p* < 0.0001).

## Results and Discussion

### Characterization of the PLT@ZrO2 Nanocomposite

**Figure 1:**
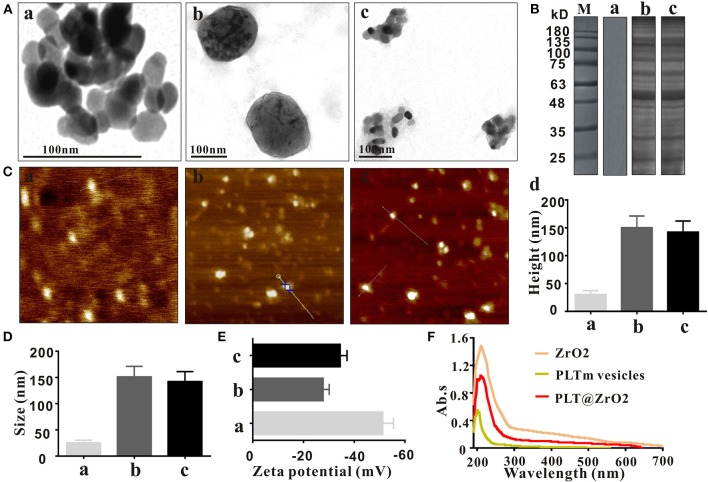
Characterization of PLT@ZrO2. **(A)** TEM images of ZrO2, PLTm vesicles, and PLT@ZrO2. Scale bar: 100 nm. a, ZrO2; b, PLTm vesicles; c, PLT@ZrO2. **(B)** SDS-PAGE protein assessment. M, Marker; a, ZrO2; b, PLTm vesicles; c, PLT@ZrO2. **(C)** The heights of ZrO2, PLTm vesicles, and PLT@ZrO2 observed under AFM. a, ZrO2; b, PLTm vesicles; c, PLT@ZrO2; d, Quantitative assay of heights of ZrO2, PLTm vesicles, and PLT@ZrO2. **(D)** The particle size of ZrO2, PLTm vesicles, and PLT@ZrO2. a, ZrO2; b, PLTm vesicles; c, PLT@ZrO2. **(E)** Zeta potential of ZrO2, PLTm vesicles, and PLT@ZrO2. a, ZrO2; b, PLTm vesicles; c, PLT@ZrO2. **(F)** UV-Vis spectra of ZrO2, PLTm vesicles, and PLT@ZrO2.

ZrO2 NPs were monodispersed with diameters averaging 25 nm (Figure 1Aa), larger than the spherical shaped ZrO2 NPs (of ~9–11 nm) extracted from E. globulus leaf (Balaji et al., 2017). The diameter of PLTm vesicles was about 150 nm (Figure 1Ab), consistent with the previous report (Shang et al., 2019). Ultrasonic treatment facilitated encapsulation of ZrO2 NPs by PLTm vesicles to form PLT@ZrO2 nanocomposites. As shown in Figure 1Ac, several ZrO2 NPs were camouflaged by one PLTm vesicle. The SDS-PAGE results (Figure 1B) indicated that the proteins of PLT@ZrO2 nanocomposites were almost the same as PLTm nanovesicles. The heights of ZrO2 NPs, PLTm nanovesicles and PLT@ZrO2 nanocomposites observed under an AFM were 30.0 ± 7.2, 150 ± 21.1, and 142 ± 20.2 nm (Figure 1C). Dynamic light scattering (DLS) data (Figure 1D) showed that the average size of PLT@ ZrO2 nanocomposites were 140 nm, slightly smaller than PLTm nanovesicles and consistent with the data from AFM. Zeta potential of ZrO2 NPs was −51.5 ± 3.1 mV. After encapsulation, Zeta potential of PLT@ZrO2 was −34.5 ± 2.7 mV, similar to that of PLTm nanovesicles (−27.8 ± 2.4 mV) (Figure 1E), indicating successful camouflage. Results from UV-vis spectrometry (Figure 1F) showed that PLT@ZrO2 possesses absorption peaks at 210 and 200 nm, consistent with those of ZrO2 NPs and PLTm nanovesicles detected alone. These findings demonstrated the successful preparation of PLT@ZrO2.

### *In vitro* Antitumor Effects of PLT@ ZrO2

#### *In vitro* Effects of PLT@ZrO2 Nanocomposite on Hela Cells Viability

**Figure 2:**
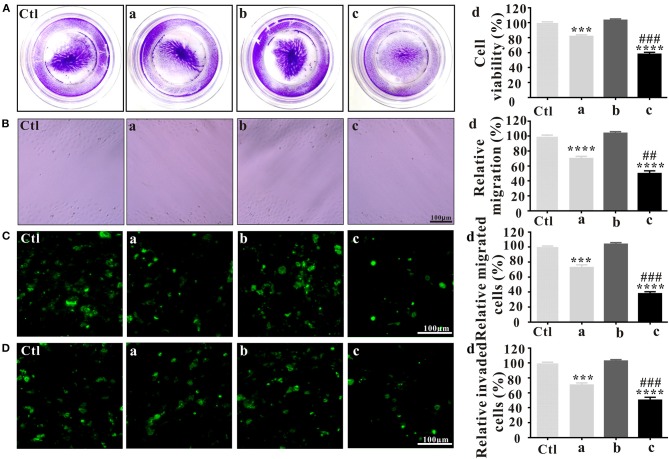
*In vitro* antitumor effects of PLT@ZrO2. **(A)** Cell viability assay after treated with ZrO2, PLTm vesicles, or PLT@ZrO2. Ctl, control; a, ZrO2; b, PLTm vesicles; c, PLT@ZrO2; d, Semi-quantitative assay of cell viability after treated with ZrO2, PLTm vesicles, or PLT@ZrO2. **(B)** Scratch-wound healing recovery assays after treated with ZrO2, PLTm vesicles, or PLT@ZrO2, respectively. Ctl, control; a, ZrO2; b, PLTm vesicles; c, PLT@ZrO2; d, Semi-quantitative assay of migration rate after treated with ZrO2, PLTm vesicles, or PLT@ZrO2. **(C)** Transwell migration assay after treated with ZrO2, PLTm vesicles, or PLT@ZrO2, respectively. Ctl, control; a, ZrO2; b, PLTm vesicles; c, PLT@ZrO2; d, Semi-quantitative assay of migration rate after treated with ZrO2, PLTm vesicles, or PLT@ZrO2. **(D)** Transwell invasion assays after treated with ZrO2, PLTm vesicles, or PLT@ZrO2, respectively. Ctl, control; a, ZrO2; b, PLTm vesicles; c, PLT@ZrO2; d, Semi-quantitative assay of invasion rate after treated with ZrO2, PLTm vesicles, or PLT@ZrO2. Data are mean ± SD (*n* = 3). Compared to the control group: ***p < 0.001 and ****p < 0.0001; compared to the ZrO2 group: ^***##***^*p* < 0.01 and ^**###**^*p* < 0.001.

Before investigating the anti-metastatic potential of PLT@ZrO2, we used crystal violet staining to assess the viability of Hela cells after treatment with ZrO2, PLTm vesicles and PLT@ZrO2. Our results showed that compared with ZrO2, PLT@ZrO2 greatly inhibited viability, indicating that PLT camouflaging enhanced the inhibitory effect of ZrO2 NPs (Figure 2A). This may be due to increased local concentration of ZrO2 when PLTm vesicles bind to tumor cells (Shang et al., 2019).

#### PLT@ZrO2 Potently Inhibits Hela Cells Migration and Invasion

In the scratch-wound healing recovery assay (Figure 2B), ZrO2 NPs partly inhibited wound healing of Hela cells. PLT@ZrO2 greatly inhibited Hela cell migration, while PLTm did not inhibit Hela cells migration.

As shown in Figure 2C, treatment with ZrO2 NPs alone inhibited the migration of Hela cells by ~30%, and PLT@ZrO2 exerted significant additional anti-migration effect. The Transwell invasion assay (Figure 2D) revealed that PLT@ZrO2 dramatically inhibited cells invading through Matrigel-coated filters, thus significantly decreasing the metastasis of Hela cells. The above results demonstrated that PLTm vesicle camouflaging improved the inhibitory effect of ZrO2 NPs on the migration and invasion of Hela cells.

### Biocompatibility of the PLT@ZrO2 Nanocomposite

**Figure 3:**
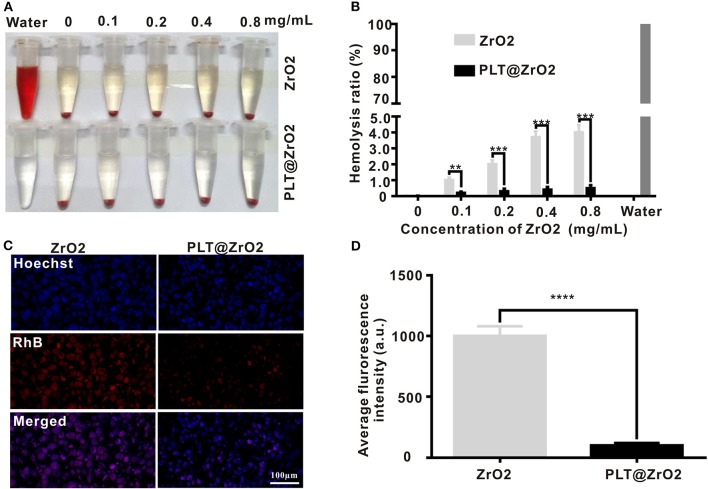
Biocompatibility of PLT@ZrO2. **(A)** Pictures of RBC suspensions after treatment with various concentrations of ZrO2 or PLT@ZrO2. **(B)** Hemolytic rate after treatment with various concentrations of ZrO2 or PLT@ZrO2. **(C)** Images of RAW264.7 cells upon culture with ZrO2-RhB or PLT@ZrO2-RhB for 24 h taken by a LCFM. Scale bar: 100 μm. **(D)** Average fluorescence intensity of RAW264.7 cells after cultured with ZrO2-RhB or PLT@ZrO2-RhB for 24 h. Data are presented as mean ± SD (*n* = 3). Compared to the ZrO2 group: **p < 0.01, ***p < 0.001, and ****p < 0.0001.

We evaluated the toxicities of ZrO2 NPs and PLT@ZrO2 by observing their hemolytic effects on RBCs. As shown in Figure 3A, the hemolytic rate after treatment with ZrO2 NPs, even in the concentration of 0.8 mg/mL, was <4.5%. PLT@ZrO2 had a value (0.5 ± 0.29%) significantly lower than that of ZrO2 NPs (Figure 3B), indicating that PLTm vesicle camouflaging improved the biosafety and hemocompatibility of ZrO2 NPs.

ZrO2 NPs and PLT@ZrO2 labeled with RhB (red fluorescent signals) were applied to detect the anti-phagocytosis effect. After incubation with ZrO2-RhB for 24 h, RAW264.7 macrophages showed strong red fluorescent signals (Figure 3C), demonstrating phagocytosis. However, weak fluorescent signals were observed after treatment with PLT@ZrO2-RhB, indicating that phagocytic activity was significantly weakened. Macrophages treated with PLT@ZrO2-RhB showed lower average fluorescence than that of cells treated with ZrO2-RhB (Figure 3D). The above results suggested that PLTm nanovesicle camouflaging prevents phagocytosis of ZrO2 NPs by macrophages, thus reducing their clearance and extending the circulation time.

The above results suggested that PLT@ZrO2 nanocomposite outperformed ZrO2 NPs in biocompatibility.

### *In vivo* Distribution of PLT@ZrO2 Through Intravenous Injection

**Figure 4:**
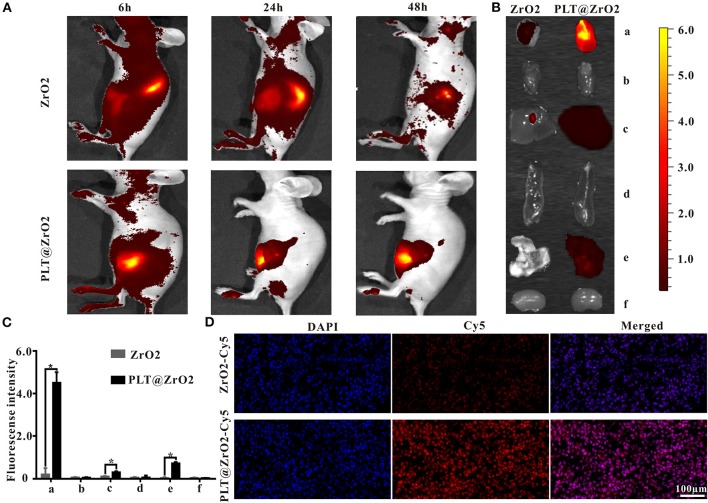
*In vivo* distribution of PLT@ZrO2 through intravenous injection. **(A)** *In vivo* fluorescent images of nude mice at 6, 24, and 48 h upon intravenous treatment with ZrO2-Cy5 or PLT@ZrO2-Cy5. **(B)** Bioluminescent images of tumors and visceral tissues at 48 h post-treatment with ZrO2-Cy5 or PLT@ZrO2-Cy5. **(C)** Semi-quantitative assay of fluorescent intensities of tumor and other tissue samples. a, Tumor; b, heart; c, liver; d, spleen; e, lung; f, kidney. **(D)** Fluorescent images of tumors from nude mice 48 h upon administration of ZrO2-Cy5 or PLT@ZrO2-Cy5. Scale bar: 100 μm. Data are presented as the mean ± SD (*n* = 3). Compared to the ZrO2 group: *p < 0.05.

Due to the ability of PLTm vesicles to escape phagocytosis by immune cells and bind to tumor cells (Shang et al., 2019), PLT@ZrO2 has the potential to accumulate in tumors that have metastasized. To verify this hypothesis, we assessed biodistribution of PLT@ZrO2 using PLT@ZrO2 conjugated by Cy5. We used ZrO2 NPs conjugated by Cy5 as a control. As shown in Figure 4A, after administration of PLT@ZrO2-Cy5, we observed increased fluorescent intensity at tumor sites compared to ZrO2-Cy5, demonstrating increased retention of PLT@ZrO2. At 48 h after intravenous injection, PLT@ZrO2-Cy5 retentions in tumor, liver and lungs were greater than those of ZrO2-Cy5 (*P* < 0.05) (Figures 4B,C), and more intense red fluorescence in tumor tissue in the PLT@ZrO2 group was observed (Figure 4D), suggesting that retention of PLT@ZrO2-Cy5 in tumor sites exceeded that of ZrO2-Cy5 and PLT@ZrO2 nanocomposites have excellent tumor targeting efficiency.

### Antitumor Effects of PLT@ZrO2 *In vivo*

**Figure 5:**
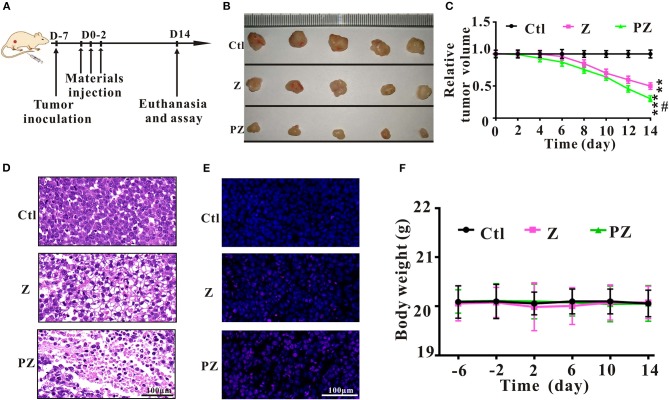
Anticancer effects of ZrO2 and PLT@ZrO2 on Hela cells bearing BALB/c nude mice. **(A)** The scheme of experiment process. **(B)** The representative picture of tumors after treated with ZrO2 or PLT@ZrO2. **(C)** The changes of tumor volume after treated with ZrO2 or PLT@ZrO2. **(D)** The representative cell morphology of tumor tissues after treated with ZrO2 or PLT@ZrO2, and HE staining. **(E)** TUNEL assay after treated with ZrO2 or PLT@ZrO2. **(F)** Changes of body weight after treated with ZrO2 or PLT@ZrO2. Ctl, Control; Z, ZrO2; PZ, PLT@ZrO2. Scale bar: 100 μm. Data are presented as the mean ± SD (*n* = 3). Compared to the control group: **p < 0.01 and ***p < 0.001; compared to the ZrO2 group: ^#^*p* < 0.05.

To further explore the anticancer effect of PLT@ZrO2, we conducted *in vivo* assays in tumor-bearing mice that underwent Hela cells injection (Figure 5A). Compared to the ZrO2 group, tumor volumes in the PLT@ZrO2 group were smaller (Figures 5B,C), indicating that PLT@ZrO2 significantly inhibited tumor growth. As shown by HE (Figure 5D) and TUNEL staining (Figure 5E), we observed more necrotic and apoptotic cells in tumor tissues with PLT@ZrO2 compared to ZrO2. The above findings demonstrate that PLT@ZrO2 induced stronger anticancer effects than ZrO2 alone. The body weights of mice in PLT@ZrO2 group did not decline, indicating no obvious systemic toxicity (Figure 5F).

Tumor metastasis, an important feature of malignant tumors, is a complex and multistep process, regulated by genetic, and epigenetic changes (Gupta and Massagué, 2006; Tiwari et al., 2013). It is recognized that epithelial-mesenchymal transition (EMT) plays an important role in invasion and metastasis (van Zijl et al., 2009). EMT initiates the early steps of tumor metastasis and spread of tumor cells by endowing them greater motility and invasiveness (Thiery et al., 2009).

**Figure 6:**
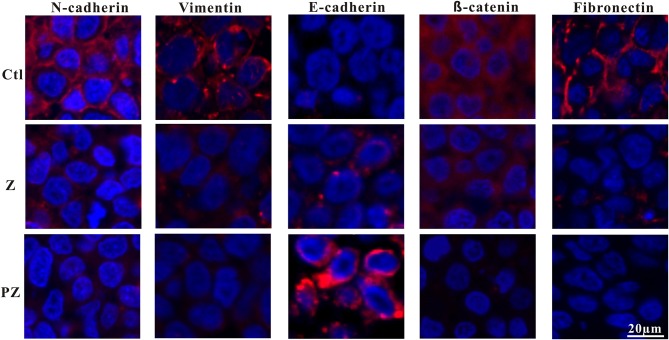
Immunofluorescence pictures of tumor sections after treated with ZrO2 or PLT@ZrO2 and stained with N-cadherin, Vimentin, E-cadherin, β-catenin, and Fibronectin. Ctl, Control; Z, ZrO2; PZ, PLT@ZrO2. Scale bar: 20 μm.

Wnt/β-catenin signaling plays a vital role in accelerating the process of EMT and metastasis (Zhou et al., 2016; Liu et al., 2017). Constitutive activation of the Wnt/β-catenin pathway results in the reduction of E-cadherin and induction of EMT (Goto et al., 2017), thereby enabling metastasis and invasion by reducing cell-to-cell contact (Onder et al., 2008). Moreover, downregulation of E-cadherin followed by EMT plays an important role in metastasis initiation (Shu et al., 2019). Previous studies have demonstrated that upregulation of N-cadherin is associated with tumor invasion and metastasis (Watson-Hurst and Becker, 2006; Hao et al., 2012; Mrozik et al., 2018). As shown in Figure 6, compared to ZrO2 NPs alone, treatment with PLT@ZrO2 downregulated N-cadherin and β-catenin (red fluorescence), and upregulated E-cadherin (red fluorescence), indicating that PLT@ZrO2 potentially inhibits tumor invasion and metastasis.

Reduction of epithelial proteins, such as E-cadherin (Onder et al., 2008), and increase of mesenchymal proteins, such as vimentin (Huber et al., 2005), are hallmarks of EMT. Vimentin, a 57 kDa type III intermediate filament protein, is critical for cell adhesion, migration, and signaling (Ivaska et al., 2007), and is essential to the progression and prognosis of cancer through EMT (Gugnoni et al., 2016; Sun and Fang, 2016). Fibronectin, a tumor-associated extracellular matrix protein, facilitates polymerization of fibrillar components on adherent and suspended tumor cell surfaces and maintains structure and motility in cell migration (Cheng et al., 1998; Huang et al., 2008; Shi et al., 2010; Knowles et al., 2015). As shown in Figure 6, compared with ZrO2 NPs, treatment with PLT@ZrO2, reduced Fibronectin and Vimentin (red fluorescence), indicating that PLT@ZrO2 could significantly inhibit tumor invasion and metastasis.

The EMT program is regulated by 3 EMT-inducing transcription factors families: Snail, Twist and Zeb (Ansieau et al., 2014; Puisieux et al., 2014). Slug (termed Snail2), can inhibit E-cadherin expression and promote EMT (Wang et al., 2009). Previous study has demonstrated that Slug regulates malignant transformation and metastasis of various cancers (Alves et al., 2009). WNT, TGF-β, NOTCH, and SHH signaling pathways play crucial roles in activation of EMT-related transcription factors, including Snail, Slug, Zeb1/2, and Twist (Nieszporek et al., 2019).

**Figure 7:**
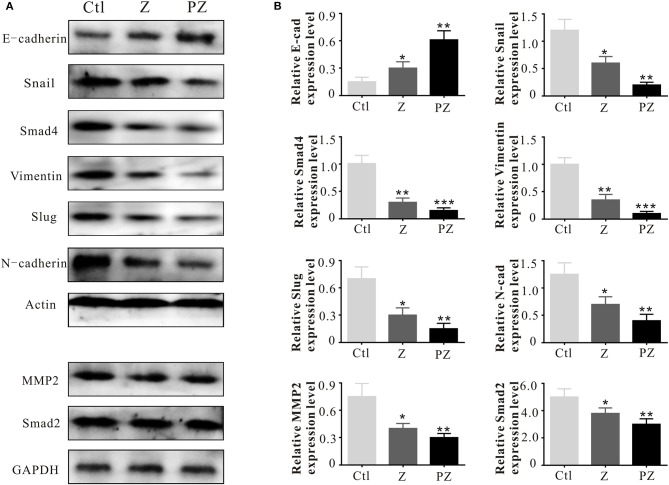
Invasion and metastasis associated protein expressions. **(A)** Invasion and metastasis associated protein expressions in tumor tissues at 14th day after intravenous injection of ZrO2 or PLT@ZrO2. **(B)** Semi-quantitative assay of invasion and metastasis associated protein expressions in tumor tissues at 14th day after intravenous injection of ZrO2 or PLT@ZrO2. Ctl, Control; Z, ZrO2; PZ, PLT@ZrO2. Data are mean ± SD (*n* = 3). Compared to the control group: *p < 0.05, **p < 0.01, and ***p < 0.001.

The adhesion molecule, N-cadherin, is related to invasive ability in cancers, and its overexpression facilitates motility and invasion (Nakajima et al., 2004). Slug and Snail inhibit E-cadherin expression; thus their overexpression promotes EMT (Hotz et al., 2007; Grzegrzolka et al., 2015). As shown in Figure 7, compared with ZrO2 NPs, treatment with PLT@ZrO2 downregulated N-cadherin, Slug, and Snail and upregulated E-cadherin, indicating that PLT@ZrO2 strongly inhibited EMT.

Matrix metallopeptidases (MMPs) degrade the extracellular matrix and basement membrane (Lengyel et al., 2001), enhancing the spread of cancer cells to distant sites (Shen et al., 2017). MMP2 and MMP9 degrade collagen IV in the extracellular basement membrane (Roomi et al., 2010) and rearrange the extracellular matrix during invasion and migration of cancer cells (Nabeshima et al., 2002; Alaseem et al., 2019). Moreover, active level of MMP2 in cancer cells is related to invasion and metastasis (Celentano et al., 2020). As shown in Figure 7, compared with ZrO2 NPs, treatment with PLT@ZrO2, significantly downregulated MMP2.

Vimentin promotes the stemness of cancer cells by phosphorylation of Slug to initiate EMT (Virtakoivu et al., 2015). Additionally, Vimentin is essential for membrane localization and appropriate activation of MT1-MMP, which is essential for endothelial sprouting (Kwak et al., 2012). As shown in Figure 7, Vimentin was down-regulated after treatment with PLT@ZrO2, indicating inhibition of EMT initiation.

The TGF-β1 receptor, Smad, forms a heteromeric complex with Smad2 and Smad4. The activated Smad factor enters the nucleus and regulates the transcription of target genes to promote cell biological behaviors, such as proliferation, invasion, and EMT (Liu et al., 2016; Li et al., 2018). As shown in Figure 7, compared with ZrO2 NPs, after treated with PLT@ZrO2, Smad2, and Smad4 were downregulated, indicating that PLT@ZrO2 strongly inhibited tumor proliferation, invasion, and EMT.

**Figure 8:**
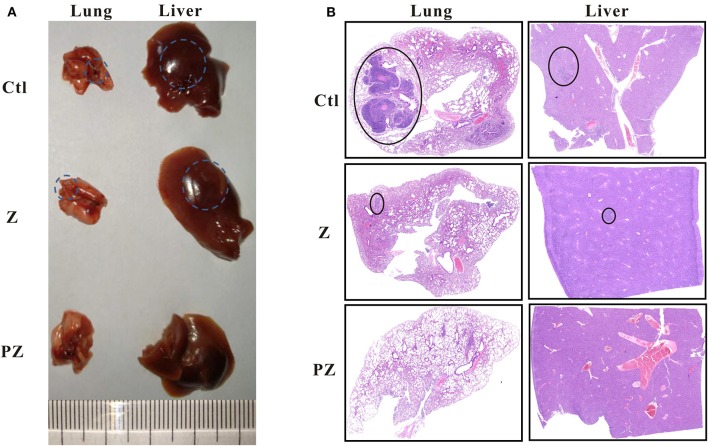
Effects of PLT@ZrO2 on lung and liver metastasis of tumor. **(A)** The representative picture of lungs and livers after treated with ZrO2 or PLT@ZrO2. **(B)** The maximum cross sections of lung and liver sections stained by HE. The circles indicate the metastatic focuses in lungs and livers. Ctl, Control; Z, ZrO2; PZ, PLT@ZrO2.

We observed lung and liver metastasis in the control and ZrO2 groups, while metastasis in the PLT@ZrO2 group was absent (Figure 8A). The maximum cross sections of lungs and livers stained by HE showed that the size of lungs and livers metastasis foci were larger in the control group compared to the ZrO2 group, while no metastasis foci were found in the sections of lungs and livers in the PLT@ZrO2 group (Figure 8B), indicating that PLT@ZrO2 could inhibit metastasis to the lungs and liver.

### Toxic and Side Effects of Vital Tissues and Organs

**Figure 9:**
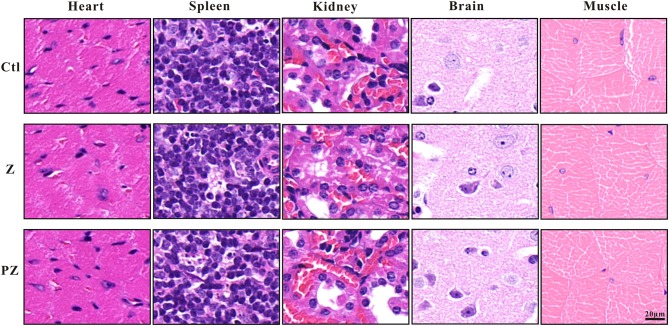
Histological images of heart, spleen, kidney, brain and skeletal muscle after ZrO2 or PLT@ZrO2 treatment and HE staining. Ctl, Control; Z, ZrO2; PZ, PLT@ZrO2. Scale bar: 20 μm.

After treatment with PLT@ZrO2, white blood cell (WBC), RBC and PLT counts did not decrease (Table 1), indicating that PLT@ZrO2 did not induce hematological toxicity. To assess the effects of PLT@ZrO2 on visceral organs, serum enzyme level detection and histological assessment were performed. As shown in Table 1, there were no alterations in blood urea nitrogen (BUN) and creatinine (Cr), indicating that PLT@ZrO2 did not result in kidney dysfunction. Due to tumor invasion and metastasis, alanine transaminase (ALT), and aspartate amino-transferase (AST) in the control group and ZrO2 group were significantly elevated, but levels in the PLT@ZrO2 group were normal, indirectly indicating that PLT@ZrO2 inhibited the liver invasion and metastasis of Hela cells. Indicators of cardiac function, such as Lactate dehydrogenase (LDH), hypersensitive troponin T (TNT-HS), creatinine kinase (CK), creatinine kinase-MB (CK-MB), and myoglobin (Myo), were not elevated in the PLT@ ZrO2 group (Table 1). Histological images of heart, spleen, and kidney did not show abnormalities in both ZrO2 and PLT@ZrO2 groups (Figure 9). These results indicated that PLT@ZrO2 could inhibit the elevation of ALT and AST and exhibit fewer side effects.

**Table 1:** The blood cell counts, the enzyme level and myocardial enzyme spectrum analysis of tumor bearing mice after treated with ZrO2, and PLT@ZrO2.

	**Control**	**ZrO2**	**PLT@ ZrO2**
**Blood cell count**			
WBC (10^9^/L)	7.4 ± 0.3	7.3 ± 0.5	7.4 ± 0.5
RBC (10^12^/L)	11.3 ± 1.2	11.3 ± 1.2	11.3 ± 1.0
HGB (g/dL)	14.6 ± 0.5	14.5 ± 0.4	14.5 ± 0.6
HCT (%)	49.2 ± 1.4	49.2 ± 1.3	49.0 ± 1.5
PLT (10^11^/L)	8.6 ± 0.4	8.5 ± 0.5	8.4 ± 0.6
**Serum enzyme level**			
ALT (U/L)	25.5 ± 2.4↑	19.8 ± 1.8↑	9.5 ± 0.6
AST (U/L)	108.8 ± 2.3↑	97.9 ± 2.2↑	48.2 ± 2.4
Urea (mmol/L)	2.1 ± 0.2	2.1 ± 0.2	2.2 ± 0.3
CRE (μmol/L)	6.7 ± 0.3	6.7 ± 0.4	6.8 ± 0.4
**Myocardial enzyme spectrum**			
TNT-HS (pg/ml)	48.7 ± 3.6	48.4 ± 3.7	48.8 ± 4.1
CK (U/L)	3780.0 ± 121.5	3785.3 ± 127.1	3791.4 ± 125.8
LDH-L (U/L)	7690.5 ± 120.2	7692.5 ± 122.3	7700.4 ± 131.8
CK-MB (U/L)	3770.9 ± 86.3	3782.7 ± 103.4	3792.9 ± 115.1
Myo (ng/mL)	72.9 ± 2.9	73.6 ± 2.5	73.9 ± 3.5

*Data are mean ± SD (n = 3)*.

## Conclusion

In this study, we have shown PLT@ZrO2, a PLTm vesicle-encapsulated ZrO2 nanocomposite, targeted subcutaneous and metastatic tumor sites, enhanced the anticancer effects of ZrO2, and inhibited tumor invasion and metastasis. ZrO2 NPs coated with PLTm vesicles escaped phagocytosis by immune cells, extended their retention, and targeted tumor sites. Additionally, we did not observe systemic toxicity after treatment with PLT@ZrO2. The PLT@ZrO2 nanocomposite has potential as a non-toxic and efficient targeted anticancer platform capable of inhibiting tumor growth, invasion and metastasis.

## Data Availability Statement

The original data supporting this article conclusions will be made available to any qualified researcher by the authors without reservation.

## Ethics Statement

The ethics committee of Third Xiangya Hospital of Central South University (No. 2017-s75) approved the animal experiment, and the guidelines released by the Ministry of Science and Technology of the People's Republic of China in September 30th, 2006 guided the animal care.

## Author Contributions

The manuscript was written through contributions of all authors. All authors have given approval to the final version of the manuscript.

## Conflict of Interest

The authors declare that the research was conducted in the absence of any commercial or financial relationships that could be construed as a potential conflict of interest.
